# Immobilizing bacteria to prevent infections

**DOI:** 10.7554/eLife.107383

**Published:** 2025-06-03

**Authors:** Patricia Reist Iscar, Petr Broz

**Affiliations:** 1 Department of Immunobiology, University of Lausanne Lausanne Switzerland

**Keywords:** guanylate-binding proteins, coatomer, actin tail, Burholderia thailandensis, Shigella flexneri, GVIN1, Other

## Abstract

The interferon-induced GTPase GVIN1 prevents bacteria from spreading among cells by coating them and rendering them immobile.

**Related research article** Guo W, Apte SS, Dickinson MS, Kim SY, Kutsch M, Coers J. 2025. Human giant GTPase GVIN1 forms an antimicrobial coatomer around the intracellular bacterial pathogen *Burkholderia thailandensis*. *eLife* **14**:RP106896. doi: 10.7554/eLife.106896.

Bacteria have evolved sophisticated strategies to invade host cells and replicate inside them while avoiding detection by the immune system. Some species of bacteria, such as *Shigella* and *Burkholderia,* can hijack a key protein in the host cell’s cytoskeleton called actin. These bacteria use specific proteins known as IcsA and BimA to bind actin – which is involved in cell-cargo transport and movement – and build tail-like structures that propel them rapidly within cells. This also allows them to enter neighboring cells directly, which reduces their chances of being detected (Lamason and Welch, 2017).

In turn, host cells have developed several mechanisms to prevent intracellular bacteria from replicating and spreading. For instance, guanylate-binding proteins or GBPs, a group of the GTPase protein family that are transcriptionally induced by immune signals known as interferons, can recognize and target intracellular bacteria (Kim et al., 2016). They achieve this by binding to components of the bacterial cell membrane called lipopolysaccharides, and covering their surface with protein complexes known as GBP coats or coatomers (Kutsch et al., 2020; Santos et al., 2020; Wandel et al., 2020). These coatomers have also been shown to restrict the motility of some bacteria by preventing them from forming actin tails (Piro et al., 2017; Wandel et al., 2017). However, the role of other members of the GTPase family in this antimicrobial defense strategy remained unknown. Now, in eLife, Jörn Coers and colleagues from Duke University Medical Center – including Weilun Guo as first author – report that a ‘giant GTPase’ known as GVIN1 also stops some bacteria from spreading by forming coatomers (Guo et al., 2025).

The team began by studying *Burholderia thailandensis* in human cell lines grown in the laboratory. They found that although a GBP known as GBP1 formed coatomers on *B. thailandensis* in several different cell lines, some bacteria could still form actin tails. They discovered that some cell types, such as the T24 cells, expressed a crucial cofactor protein of GBP1, which was required to restrict tail formation. In contrast, other cell lines, such as HeLa cells, lacked this cofactor and actin tails could still form. Guo et al. further observed that treating T24 cells with interferon gamma (IFNγ) abolished tail formation in *B. thailandensis* (Figure 1). However, when the gene for GBP1 was deleted, tail formation was only partially restored, indicating that T24 cells must possess another IFNγ-inducible factor that contributes to blocking this process.

**Figure 1.**
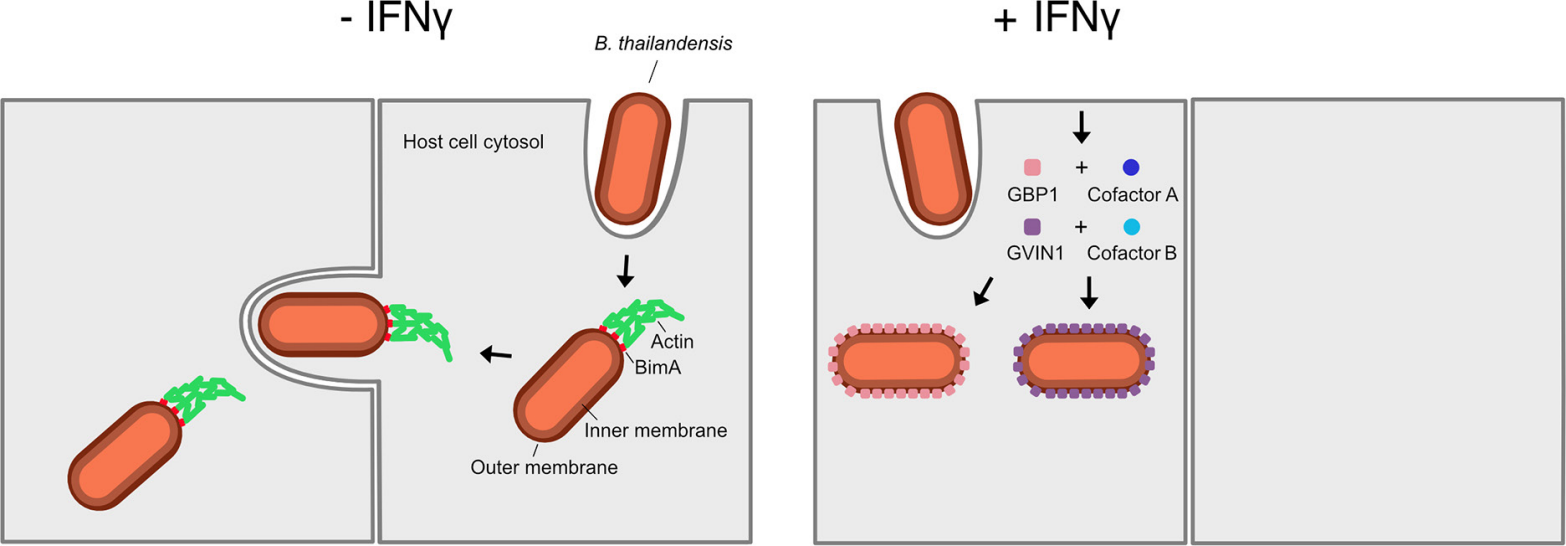
Cells use coatomers to restrict the movement of bacteria from cell to cell. Left: Without the immune signaling molecule interferon gamma (IFNγ), *Burkholderia thailandensis* bacteria (red rods) use the protein BimA (red squares) to assemble actin (green) from the host cell to form a tail-like structure that allows them to move and spread to neighboring cells. Right: Treating cells with IFNγ upregulates the GTPases GBP1 (pink squares) and GVIN1 (purple squares), which form coatomers on the surface of *B. thailandensis*, restricting their ability to source actin from their host and consequently their ability to infect other cells. The activity of both GTPases depends on cofactors (blue circles) that have yet to be identified.

To identify this additional factor, Guo et al. used RNA sequencing and small interfering RNAs to reveal the gene activity required for tail formation in IFNγ-treated cells. This approach highlighted the GTPase GVIN1 as a leading candidate. Deleting the gene for GVIN1 in T24 cells partially restored actin tail formation, similar to GBP1, and deleting both GTPases completely restored tail formation. Further experiments confirmed that GVIN1 formed coatomers on the bacteria, which resulted in the loss of BimA and consequent tail assembly. Together, these findings indicate that GVIN1 acts independently of GBP1 to inhibit actin tail formation by *B. thailandensis*.

Next, they identified similarities and differences in how GBP1 and GVIN1 restrict actin tail formation in *B. thailandensis*. While both GBP1 and GVIN1 appear to require additional cofactors to limit bacterial motility, only GVIN1 requires O-antigen – a major component of lipopolysaccharide – to restrict tail formation. Additionally, the proteins appear to recognize different pathogens: experiments with *Shigella flexneri* revealed that actin tail formation was solely restricted by GBP1, whereas *B. thailandensis* was targeted by both GVIN1 and GBP1.

The study conducted by Guo et al. marks a significant milestone by demonstrating an antimicrobial function for GVIN1, paving the way for further research into coatomer-forming GTPases and how individual cells defend themselves from infection. In future studies, it will be essential to identify the cofactors required for GBP1 and GVIN1 to inhibit actin tail formation and to determine whether these are specific to certain bacterial species. Additionally, it is important to investigate whether these cofactors are involved in other immune processes, such as inflammasome activation. Finally, it is still unclear whether GVIN coatomers restrict bacterial cell-to-cell spread and if they can effectively contain infections in animal models.

In summary, this study highlights the immune system’s diverse and surprising arsenal of antimicrobial effectors, some of which are specifically designed to target intracellular bacteria and prevent their spread. Further investigation into interferon-induced proteins may reveal unexpected mechanisms and lead to new therapies for intracellular infections.
